# Mathematical modelling of synergistic triple-antibiotic therapy against carbapenem-resistant Acinetobacter baumannii at clinically achievable concentrations

**DOI:** 10.1099/jmm.0.002086

**Published:** 2025-11-05

**Authors:** Tuan Huynh, Thanh Lam, Ngan Pham, Loan Luong, My Dung Jusselme

**Affiliations:** 1 Department of Microbiology and Parasitology, University of Medicine and Pharmacy, 217 Hong Bang Street, Ward Cho Lon, Ho Chi Minh City, Vietnam; 2 Department of Infection Control, University Medical Center, 215 Hong Bang Street, Ward Cho Lon, Ho Chi Minh City, Vietnam; 3 Department of Microbiology, University Medical Center, 215 Hong Bang Street, Ward Cho Lon, Ho Chi Minh City, Vietnam; 4 Faculty of Medical Technology, Van Lang University, 69/68 Dang Thuy Tram Street, Ward Binh Loi Trung, Ho Chi Minh City, Vietnam; 5 LEESU, Univ Paris Est Creteil, ENPC, Institut Polytechnique de Paris, Creteil, France

**Keywords:** *Acinetobacter baumannii*, antibiotic synergy, carbapenem resistance, mathematical modelling, triple combination therapy

## Abstract

**Introduction.** Carbapenem-resistant *Acinetobacter baumannii* (CRAB) represents a critical healthcare threat with limited treatment options, particularly challenging in Southeast Asia where resistance rates exceed 65%.

**Hypothesis/Gap Statement.** Current synergy testing methods are labour-intensive and poorly standardized, limiting their clinical application. We hypothesize that synergistic interactions between antibiotics follow predictable mathematical patterns derivable from separate MIC determinations for each antibiotic.

**Aim.** To evaluate the efficacy of colistin-meropenem-ampicillin/sulbactam combinations at standard dosages and develop a predictive mathematical model for synergistic interactions against CRAB.

**Methodology.** A cross-sectional study was conducted using 61 CRAB clinical isolates from a Vietnamese tertiary hospital. MICs were determined using broth microdilution, and synergy was assessed via checkerboard assays. Mathematical models were developed to predict fractional inhibitory concentration (FIC) values from separate MIC determinations for each antibiotic.

**Results.** All isolates demonstrated high-level meropenem resistance (MICs 32-≥128 µg ml^−1^) and ampicillin/sulbactam resistance (98.4% with MICs≥64/32 µg ml^−1^) but remained intermediate to colistin (MICs 0.0625–0.25 µg ml^−1^). The triple combination achieved 100% synergy at standard ampicillin/sulbactam doses (8/4 µg ml^−1^). Our log-transformed power model accurately predicted synergistic interactions (*R*²=0.928) using the equation log(FIC) = −2.52+(−1.02) × 1/√Mero+0.43×1/√Col+4.32×1/√As.

**Conclusion.** The triple combination achieves universal synergy at standard dosages, potentially reducing toxicity risks. Our mathematical model enables rapid prediction of effective combinations from routine susceptibility tests, offering a transformative approach to optimizing therapy against multidrug-resistant pathogens.

## Introduction

Antimicrobial resistance represents one of the most urgent global health crises of the 21^st^ century, with carbapenem-resistant *Acinetobacter baumannii* (CRAB) highlighted as a critical-priority pathogen by the World Health Organization [1]. Globally, CRAB has emerged as a major cause of healthcare-associated infections with resistance rates reaching 40–70% in many countries and mortality rates ranging from 30% to 75% in infected patients [2,3]. In high-burden regions like Southeast Asia, CRAB prevalence exceeds 65%, with attributable mortality rates of 42–52% [4,5]. In Vietnam, the situation has escalated dramatically, with studies revealing carbapenem resistance in *A. baumannii* ranging from 55% to as high as 90% [5,7]. This trend is particularly concerning in critical care settings, where in Vietnamese tertiary hospitals, the mortality rates among patients with ventilator-associated pneumonia due to CRAB reached 52% [5], and recent data from 2019 to 2020 shows overall in-hospital mortality at 56% among patients with CRAB infections [8]. The extremely high prevalence of both carbapenem resistance (90.5%) and multidrug resistance (92%) in clinical *A. baumannii* isolates demands innovative antimicrobial therapy strategies, as conventional treatment paradigms are increasingly ineffective.

Current guidelines from the Infectious Diseases Society of America (IDSA) recommend combination therapies for CRAB infections, including polymyxin-tigecycline combinations or high-dose ampicillin-sulbactam (6–9 g day^−1^) [9]. For extensively drug-resistant (XDR) isolates, triple-drug regimens incorporating colistin, meropenem and ampicillin/sulbactam have emerged as a promising strategy. This approach leverages synergistic mechanisms: colistin-induced outer membrane disruption enhances the intracellular penetration of meropenem and sulbactam, which together target complementary penicillin-binding proteins (PBPs 1a, 1b, 2 and 3), thereby simultaneously inhibiting multiple stages of bacterial cell wall synthesis [10]. However, these high-dose regimens carry considerable risks. Nephrotoxicity occurs in 30–60% of patients treated with polymyxins, while high-dose *β*-lactams are associated with neurotoxicity and seizures in up to 15% of critically ill patients [11]. Recent clinical evidence suggests that lower doses of sulbactam, aligned with CLSI breakpoints (8/4 µg ml^−1^) may retain efficacy while significantly reducing adverse effects [12]. This hypothesis warrants rigorous investigation, as it could fundamentally alter the global risk-benefit assessment of combination therapies. Moreover, the rational selection of synergistic antibiotic combinations faces a major methodological barrier. Existing laboratory methods for synergy testing such as time-kill assays, checkerboard methods and E-tests are labour-intensive (requiring 24–72 h), poorly standardized (with kappa coefficients <0.2) and rarely available in routine clinical settings [13]. As a result, clinicians often rely on empirical combinations or generalized recommendations rather than isolate-specific, evidence-based strategies.

The molecular rationale for synergy between these three antibiotics is particularly compelling. Colistin, a cationic polypeptide, competitively displaces divalent cations (Ca²^+^ and Mg²^+^) from LPS molecules in the outer membrane of Gram-negative bacteria, disrupting membrane integrity and increasing permeability [14]. This disruption enhanced the entry of meropenem, which targets PBP2, essential for maintaining bacterial cell shape. Colistin, a member of the polymyxin family, was selected for this study due to its established Clinical and Laboratory Standards Institute (CLSI) breakpoints and widespread availability in clinical laboratories. While polymyxin B may offer certain advantages in terms of side effect profile as noted in recent Infectious Diseases Society of America (IDSA) guidelines, both agents share similar mechanisms of action through outer membrane disruption. The mathematical modelling framework developed in this study could potentially be adapted for other polymyxin agents, including polymyxin B, once appropriate validation studies are completed. Concurrently, sulbactam exhibits intrinsic antimicrobial activity against *A. baumannii* via high-affinity binding to PBP1a/1b and PBP3, which are critical for cell division processes [15]. The simultaneous inhibition of these complementary targets provides a mechanistic basis for the synergy observed in preliminary studies. We hypothesize that synergistic interactions between antibiotics against *A. baumannii* follow predictable mathematical patterns that can be derived from separate MIC determinations for each antibiotic. This represents a paradigm shift from traditional synergy testing, enabling precise prediction of antibiotic interactions without additional *in vitro* procedures. Our objectives are twofold: (i) to assess the *in vitro* efficacy of colistin, meropenem and ampicillin/sulbactam, both individually and in combination, against CRAB clinical isolates, and (ii) to develop and validate a novel predictive mathematical model for three-drug synergistic interactions based on the separate MIC determinations for each antibiotic. This approach has the potential to transform antimicrobial stewardship by enabling rapid, accurate prediction of effective antibiotic combinations from routinely performed susceptibility tests. Beyond *A. baumannii*, the mathematical principles established here could provide a framework for rational combination therapy design against other critical multidrug-resistant (MDR) pathogens, addressing a fundamental limitation in current infectious diseases management.

## Methods

### Clinical sample collection

A cross-sectional study was conducted at University Medical Center Ho Chi Minh City, Vietnam, between June and December 2022. Clinical specimens including sputum, blood, urine, wound swabs, cerebrospinal fluid and bronchoalveolar lavage were collected following standard hospital protocols using aseptic techniques. Blood cultures were obtained using a strict protocol of site preparation (sequential application of 2% chlorhexidine followed by 70% isopropyl alcohol for 30 s each, allowing complete drying between applications) with collection in BD BACTEC aerobic and anaerobic bottles (8–10 ml per bottle). Sputum samples were collected in sterile screw-capped containers after proper patient instruction on deep expectoration techniques to minimize salivary contamination. Respiratory samples underwent quality assessment using the Q-score system (accepted if >25 polymorphonuclear cells and <10 epithelial cells per low-power field). Wound specimens were collected using the Levine technique, rotating a sterile swab with sufficient pressure to express fluid from the wound bed. All specimens were stored at 4 °C in an automated temperature-monitored refrigeration unit and transported to the laboratory within 2 h [16].

Patient data management employed a custom-designed REDCap (Research Electronic Data Capture) database hosted on secure hospital servers with 256-bit encryption and role-based access controls. The database architecture included distinct modules for: (i) patient demographics (age, gender and hospital location), (ii) clinical parameters (primary diagnosis, comorbidities, ventilation status and prior antimicrobial therapy), (iii) laboratory data (specimen type, collection date/time and culture results) and (iv) antimicrobial susceptibility findings. Data validation rules were embedded to ensure accuracy, including range checks, logical rules and required field completion. Daily automated backup protocols were established, and all data access was logged for regulatory compliance. Access to the complete dataset was restricted to the principal investigator and designated research team members.

### Collection of *A. baumannii* isolates and morphological identification

Clinical specimens were cultured on both MacConkey agar (BD Difco™) and sheep blood (5%) supplemented trypticase soy agar (BD BBL™) using a standardized quadrant streak technique to obtain isolated colonies. Plates were incubated aerobically in a microprocessor-controlled incubator at 37 °C (±0.5 °C) for 24–48 h with humidity maintenance at 85–90%. Colonies with morphological features consistent with *A. baumannii* were carefully selected for further analysis. These characteristically appeared as non-lactose fermenting, smooth, slightly mucoid, grayish-white colonies of 1–2 mm diameter on MacConkey agar after 24 h and as non-haemolytic, dome-shaped colonies on blood agar with no distinctive odour.

Preliminary identification involved Gram staining revealing Gram-negative coccobacilli, often appearing as diplococci in clinical specimens and short rods in culture, with negative oxidase testing using 1% tetramethyl-p-phenylenediamine dihydrochloride and positive catalase reaction with 3% hydrogen peroxide. Colony morphology was examined both macroscopically and with a stereomicroscope at 40× magnification to confirm typical features.

Species identification was conducted utilizing the BD Phoenix™ Automated Microbiology System (BD Diagnostic Systems, Sparks, MD, USA) with the NMIC/ID-92 panel following manufacturer guidelines [17]. The Phoenix™ platform employs various colorimetric and fluorometric biochemical assays coupled with algorithmic analysis to differentiate bacterial species. Bacterial suspensions were adjusted to a 0.5–0.6 McFarland turbidity using a calibrated nephelometer. Daily quality assurance was implemented using *Escherichia coli* ATCC 25922. For specimens with Phoenix identification confidence scores lower than the manufacturer’s recommended threshold (≥90%), supplementary confirmatory biochemical testing was conducted, including triple sugar iron agar reaction patterns, citrate metabolism on Simmons citrate agar and growth evaluation in nutrient broth at 42 °C for 24 h. *A. baumannii* ATCC 19606 served as the reference isolate throughout all identification protocols.

During the study period, we collected 53 non-duplicate *A. baumannii* isolates from 53 unique patients. Non-duplicate isolates were defined as the first isolate of a species per patient, except when isolates from the same patient showed different antimicrobial susceptibility patterns based on interpretive category discrepancies for at least two major antimicrobial agents. Confirmed isolates were subcultured onto blood agar, incubated for 24 h at 37 °C and then preserved in 20% glycerol broth at −20 °C in cryovials with secure, colour-coded caps and dual identification labelling for subsequent analyses.

### Antibiotic susceptibility testing of MDR for *A. baumannii* isolates

Antimicrobial resistance categories were defined according to international standardized definitions proposed by Magiorakos *et al*. [18]. The MDR profile of *A. baumannii* isolates was determined by assessing non-susceptibility to at least one agent in three or more antimicrobial categories. XDR isolates were defined as those demonstrating resistance to all but ≤2 antimicrobial categories, while retaining susceptibility to ≤2 groups. Pandrug-resistant (PDR) isolates were defined as those non-susceptible to all antimicrobial agents in all categories. CRAB was defined as isolates demonstrating resistance to carbapenems (imipenem and meropenem) with inhibition zones ≤18 and ≤14 mm, respectively, using disc diffusion method according to CLSI guidelines [18].

For antimicrobial susceptibility testing, isolates were retrieved from frozen storage and subcultured twice on blood agar plates at 24-h intervals to ensure viability and purity for testing. Initial screening for carbapenem resistance was performed using the disc diffusion method with imipenem (10 µg) and meropenem (10 µg) discs (BD BBL™, USA) on Mueller–Hinton agar according to CLSI guidelines [19]. Isolates showing imipenem and meropenem inhibition zones ≤18 and ≤14 mm, respectively, were classified as carbapenem-resistant and selected for further analysis. Quality control was performed using *Pseudomonas aeruginosa* ATCC 27853 and *E. coli* ATCC 25922.

Sixty-one carbapenem-resistant *A. baumannii* isolates were identified and subjected to comprehensive antimicrobial susceptibility testing using broth microdilution to determine MICs for meropenem, colistin and ampicillin/sulbactam according to CLSI methodology [19].

### Susceptibility testing using the microdilution tray method

Antimicrobial susceptibility testing was performed using standardized broth microdilution methods in accordance with CLSI guideline. Stock solutions of meropenem trihydrate (≥98%, Sigma-Aldrich), colistin sulphate (>19,000 units/mg, Duchefa), and ampicillin sodium (>91%, Duchefa, CAS 69-52-3) with sulbactam (Sigma-Aldrich) were prepared according to CLSI 2023 recommendations [19].

The potency of each antimicrobial agent was calculated based on manufacturer-provided assay purity data. An analytical balance (Mettler Toledo, precision ±0.1 mg) was used to accurately weigh the appropriate quantities of each powder to prepare stock solutions at 1,000 µg ml^−1^, representing ten times the highest concentration needed for testing. Meropenem and ampicillin/sulbactam were dissolved in sterile, endotoxin-free distilled water, while colistin was dissolved in sterile distilled water containing 0.2% polysorbate 80 to facilitate dissolution. Small aliquots (1 ml) of each solution were prepared in sterile polypropylene microcentrifuge tubes to ensure stability and stored at −70 °C for up to 6 months or at −20 °C for up to 30 days.

For the checkerboard assay, stock solutions of meropenem (1,024 µg ml^−1^) and colistin (16 µg ml^−1^) were prepared in cation-adjusted Mueller–Hinton broth (CAMHB, HiMedia, India). Twofold serial dilutions were performed for each antibiotic in sterile 96-well microtitre plates, with meropenem concentrations ranging from 512 to 0.5 µg ml^−1^ (512, 256, 128, 64, 32, 16, 8, 4, 2, 1 and 0.5 µg ml^−1^) and colistin concentrations from 8 to 0.125 µg ml^−1^ (8, 4, 2, 1, 0.5, 0.25 and 0.125 µg ml^−1^).

**Fig. 1.**
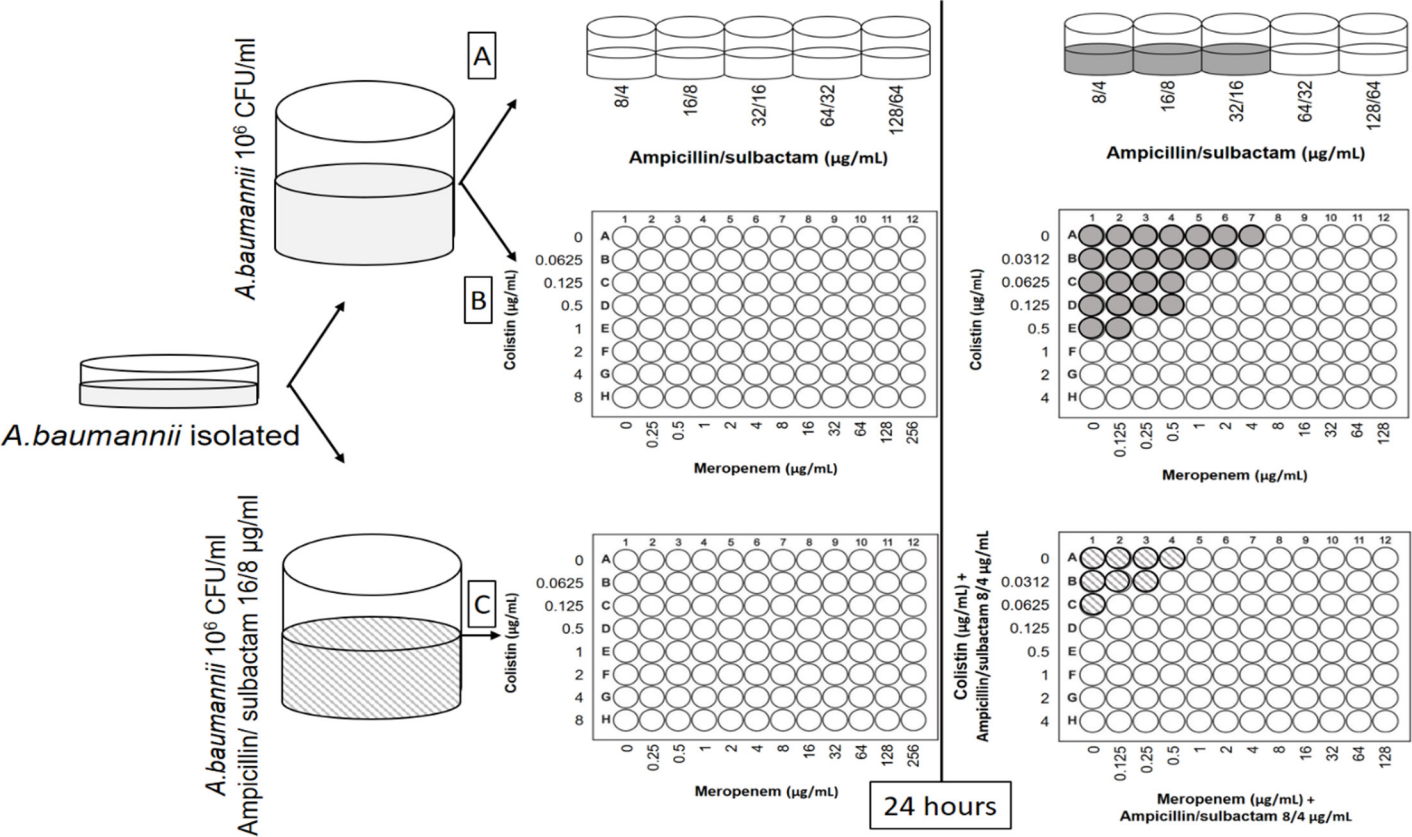
Design of susceptibility testing method.

In 96-well microtitre plates, 50 µl of each meropenem concentration was added to columns 2–12, with the highest concentration in column 2 and the lowest in column 12. Similarly, 50 µl of each colistin concentration was added to rows B–H, with row B containing the highest concentration and row H the lowest. Column 1 and row A contained 100 µl CAMHB alone as controls. Additional controls included growth control wells (no antibiotics), sterility controls (no inoculum) and single antibiotic controls for meropenem and colistin (Fig. 1).

The susceptibility testing procedure involved three sequential steps. First, bacterial suspensions were prepared by transferring 3–5 colonies from 18 to 24 h blood agar cultures to 5 ml CAMHB. The suspension was adjusted to McFarland 0.5 standard (≈1.5×10^8^ c.f.u. ml^−1^) using a calibrated densitometer. Ampicillin/sulbactam concentrations ranging from 8/4 to 256/128 µg ml^−1^ were prepared, and MIC_ampicillin/sulbactam_ was determined. Second, fresh bacterial suspensions were prepared as described above, diluted 1 : 150 in CAMHB to achieve 8–10×10^5^ c.f.u. ml^−1^, and 0.1 ml of the diluted suspension was added to each well containing various concentrations of colistin (0.0312–0.25 µg ml^−1^) and meropenem (0.125–128 µg ml^−1^). After incubation at 35–37 °C for 18–24 h, MIC_colistin_, MIC_meropenem_ and fractional inhibitory concentration (FIC)_colistin–meropenem_ were determined. Third, the procedure was repeated with bacterial suspensions diluted 1 : 150 in CAMHB containing ampicillin/sulbactam at 16/8 µg ml^−1^. After adding 0.1 ml to wells containing colistin and meropenem at the previously described concentrations and incubating at 35–37 °C for 18–24 h, FIC_colistin–meropenem–ampicillin/sulbactam_ values were determined.

To control for information bias, the broth microdilution method strictly followed the American Society for Microbiology guidelines to ensure consistency and reproducibility of results across all samples. CLSI 2023 standards were adhered to for the dilution and storage of chemicals, ensuring the stability and accuracy of antibiotic concentrations. Pure, analytical-grade chemicals were used for all antibiotic preparations. Strict compliance with CLSI M07 ‘Methods for Dilution Antimicrobial Susceptibility Tests for Bacteria That Grow Aerobically’ was maintained throughout the study, including inoculum preparation, incubation conditions and MIC determination. Laboratory technicians performing the MIC determinations were blinded to the clinical information of the isolates to prevent observer bias.

### Calculation-resistant indices including MIC and FIC

Resistant indices were calculated including the MIC and FIC of MDR *A. baumannii* isolates against colistin, meropenem and ampicillin/sulbactam when used individually, in two drugs and in a three-drug combination. MIC is defined as the lowest concentration of an antibiotic that can inhibit the growth of bacteria at a visible level. It is read as the lowest concentration at which no bacteria grow in the well (no turbidity or precipitation). The interpretation of meropenem, colistin and ampicillin/sulbactam was based on CLSI M100 ED 33 [19].

FIC is an index used to determine the synergy of drug combination formulas [20]. FIC is calculated according to the following formulas:

For two-drug combinations:


$$FIC2\;drugs=\frac{MICA\;alone}{MICA\;in\;combination}+\frac{MICB\;alone}{MICB\;in\;combination}$$

For three-drug combinations:


$$FIC3\;drugs=\frac{MICA\;alone}{MICA\;in\;combination}+\frac{MICB\;alone}{MICB\;in\;combination}+\frac{MICC\;alone}{MICC\;in\;combination}$$

For the combination formula of two drugs, if the FIC_2 drugs_ is <0.5, it means a synergistic effect, >0.5–1 indicates an additive effect, >1–2 means indifference and ≥2 indicates an antagonistic effect.

For the combination formula of three drugs, if the FIC_3 drugs_ is <0.8, it means a synergistic effect, >0.8–4 indicates an additive effect or indifference and ≥4 indicates an antagonistic effect.

Following the determination of the individual and combination MICs, we developed several mathematical models to predict FIC values using separate MIC determinations for each antibiotic data. The models explored included:

Log-transformed model with power features:
(13)log⁡(FIC)=β0+β1×Mero−0.5+β2×Col−0.5+β3×As−0.5(14)FIC=exp⁡(log⁡(FIC))
Log-transformed model with log features:
(11)log⁡(FIC)=β0+β1×log⁡(Mero)+β2×log⁡(Col)+β3×log⁡(As)(12)FIC=exp⁡(log⁡(FIC))
Model with inverse features:

$FIC=\beta 0+\beta 1\times \frac{1}{Mero}+\beta 2\times \frac{1}{Col}+\beta 3\times \frac{1}{As}$

Model with mixed features:

$FIC=\beta 0+\beta 1\times Mero+\beta 2\times \frac{1}{Col}+\beta 3\times As$

1. Model with log features:

FIC=β0​+β1​×log(Mero)+β2​×log(Col)+β3​×log(As)

For each model, we calculated performance metrics including *R*², root mean square error (RMSE), mean absolute error (MAE) and Pearson correlation coefficient to determine the most accurate and robust predictive approach.

### Sample size considerations and statistical analysis

The sample size was calculated using two complementary approaches. First, for the primary objective of evaluating synergistic effects in triple-drug combinations, we used the formula for estimating a proportion in a population:


$$n\geq Z_{1-\frac{\alpha }{2}}^{2}\left(1-p\right)p/d^{2}$$

With a type 1 error of 0.05, *P*=0.98, corresponding to the colistin-meropenem synergy rate reported in previous studies [21], and assuming that the synergy rate of the three drugs could be approximately equal to or higher than that of the two drugs, the estimated margin of error was *d*=0.05. The minimum sample size was calculated to be 31 XDR *A. baumannii* isolates.

For the secondary objective of developing a predictive mathematical model for FIC values, we considered established guidelines for regression analysis. According to Harrell, a minimum of 10 observations per predictor variable is required for reliable regression modelling in preliminary exploratory research. With three predictor variables (MICs of colistin, meropenem and ampicillin/sulbactam), this would require a minimum of 30 isolates. Additionally, Harrell recommends that the number of predictors should not exceed *m*≤*n*/10 in regression models to minimize the risk of overfitting, which our sample size satisfies [21].

Based on these calculations and considering the exploratory nature of our mathematical modelling component, our final sample of 61 *A*. *baumannii* isolates exceeds the minimum requirement for the primary objective (31 isolates) and provides adequate power for the preliminary development of predictive models with three variables. To maximize the reliability of our findings with the available sample size, we implemented k-fold cross-validation (*k*=5) rather than a separate validation cohort approach, allowing us to utilize the full dataset for both model development and internal validation while minimizing the risk of overfitting.

Data management and analysis were performed using Microsoft Excel for initial data organization, SPSS version 26.0 (IBM Corp., Armonk, NY) for descriptive statistics and basic analyses, and Python 3.8 with scikit-learn, stats-models and TensorFlow libraries for model development and visualization. Continuous variables are described by mean±sd. Categorical variables are described by frequency (percentage).

## Results

All 61 CRAB isolates from 53 participants analysed in this study exhibited an XDR profile, demonstrating resistance to all tested antimicrobial categories with the exception of polymyxins. According to current CLSI interpretive criteria, all isolates were classified as intermediate to colistin (MICs 0.0625–0.25 µg ml^−1^), as the ‘susceptible’ category no longer exists for this agent. No isolates met the criteria for solely MDR classification. PDR status could not be definitively determined due to the limited panel of antimicrobials tested; however, since all isolates demonstrated intermediate status to polymyxins rather than resistance, they did not fulfil the strict criteria for PDR classification.

### Patient and specimen characteristics

The study included 53 participants with a mean age of 72.3 years (Table 1). Male patients constituted the majority of the study population, accounting for 67.9%, while female patients made up 32.1%. In terms of department distribution, most patients were admitted to the Intensive Care Unit, representing 54.7%, followed by Internal Medicine with 28.3% and Surgery with 17.0%.

**Table 1.** Participant characteristics (*n*=53)

Characteristic	Frequency	Percentage (%)
Age (*n*=53)	72.3±14.8	
**Gender (*n*=53):** Male	36	67.9
**Department/unit (*n*=53)**		
Intensive Care Unit	29	54.7
Internal Medicine	15	28.3
Surgery	9	17.0
**Diagnosis (*n*=53)**		
Pneumonia	36	67.9
Sepsis	19	35.8
Wound infection	10	18.9
Other	16	29.6
**Specimen type (*n*=61)**		
Sputum	49	80.0
Blood	7	12.0
Urine	2	3.0
Other	3	5.0

Pneumonia was the most common diagnosis, affecting 67.9% of patients, followed by sepsis at 35.8% and wound infection at 18.9%. Other diagnoses accounted for 29.6% of cases. The high prevalence of pneumonia is consistent with previous reports identifying respiratory infections as the most common site for *A. baumannii* colonization and infection in hospitalized patients.

A total of 61 specimens were collected, with sputum samples being the most common at 80.0%, followed by blood samples at 12.0% and urine samples at 3.0%. The remaining 5.0% were classified as other specimen types. This distribution reflects the predominance of respiratory tract infections in our cohort, consistent with the high percentage of pneumonia diagnoses.

### MICs of individual drugs, two-drug combination and a three-drug combination with ampicillin/sulbactam at 8/4 μg ml^−1^

**Fig. 2.**
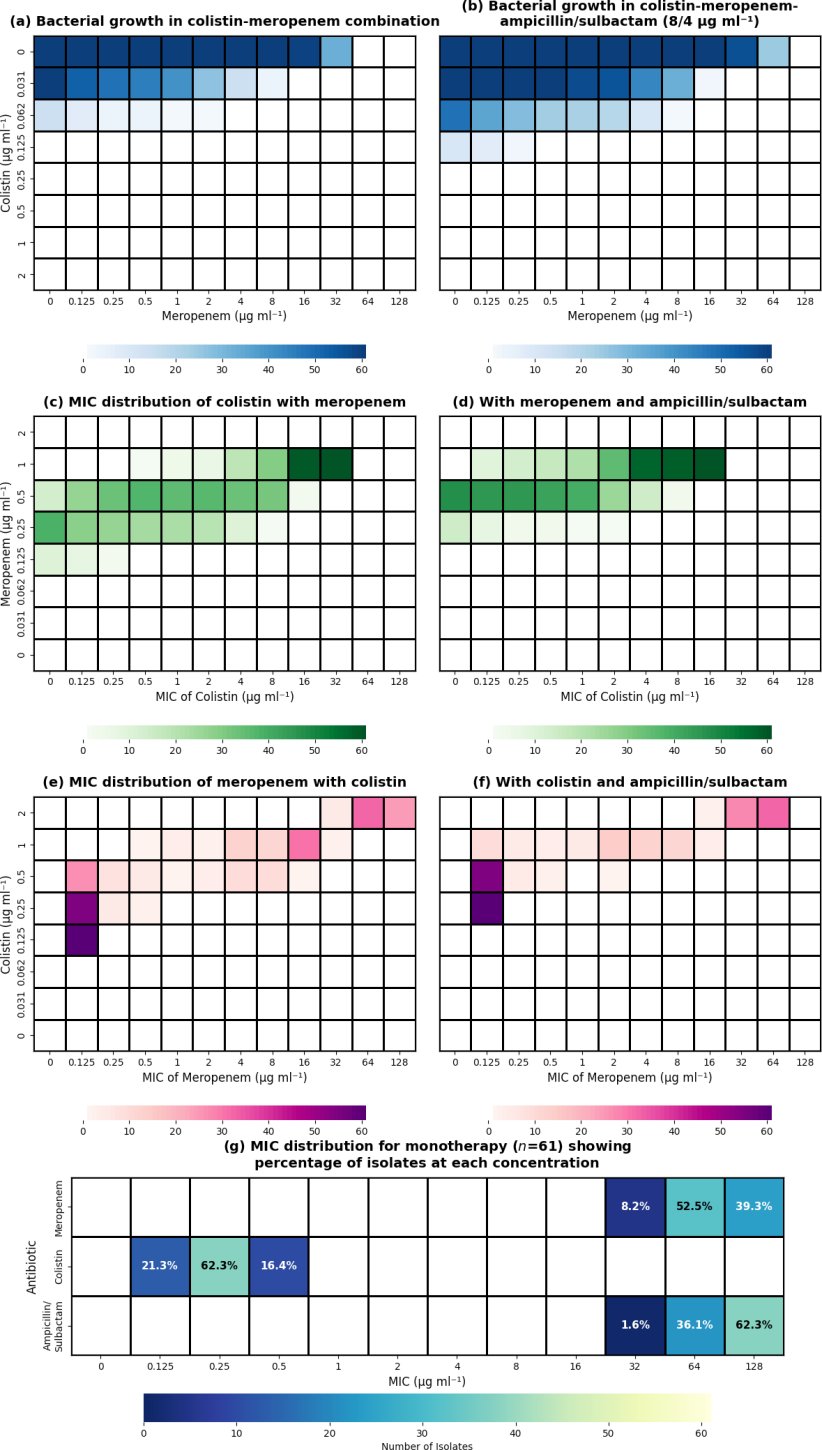
MIC distribution of individual antibiotics and their combinations against CRAB. (**a**) Bacterial growth in colistin-meropenem combination. (b) Bacterial growth in colistin-meropenem-ampicillin/sulbactam (8/4 µg/ml). (c) MIC distribution of colistin with meropenem. (d) With meropenem and ampicillin/sulbactam. (e) MIC distribution of meropenem with colistin. (f) With colistin and ampicillin/sulbactam. (g) MIC distribution for monotherapy (*n*=61) showing percentage of isolates at each concentration.

The susceptibility testing results revealed significant differences in antibiotic efficacy between monotherapies and combinations, as shown in Fig. 2. Among antibiotic monotherapies, colistin demonstrated the best activity, with most isolates being susceptible to low concentrations. The majority, 62.3%, had an MIC of 0.125 µg ml^−1^, followed by 21.3% at 0.0625 µg ml^−1^ and 16.4% at 0.25 µg ml^−1^. No isolates required higher concentrations, indicating preserved susceptibility to colistin among all tested isolates. For meropenem, most isolates exhibited high resistance, with 52.5% having an MIC of 64 µg ml^−1^, followed by 39.3% showing MIC values of ≥128 µg ml^−1^ and a smaller proportion, 8.2%, displaying an MIC of 32 µg ml^−1^. No isolates were susceptible to lower concentrations, confirming the selection of a truly carbapenem-resistant population. Ampicillin/sulbactam demonstrated poor activity as a single agent, with 62.3% of isolates requiring high MIC concentrations at ≥128/64 µg ml^−1^. Additionally, 36.1% had an MIC of 64/32 µg ml^−1^, and only 1.6% displayed susceptibility at 32/16 µg ml^−1^. No isolates were susceptible to lower concentrations, indicating widespread resistance to this combination among our isolates.

Comparison of effectiveness between two-drug (colistin+meropenem) versus three-drug (colistin+meropenem+ampicillin/sulbactam) combination against bacterial growth demonstrated different inhibition patterns. The two-drug combination inhibited bacterial growth primarily at higher concentrations, while the three-drug combination demonstrated broader inhibition across more concentration combinations. Analysis of the distribution of meropenem’s MIC showed distinct patterns in different combination therapies. When meropenem was combined with colistin, bacterial growth was concentrated at colistin levels of 0.25 µg/ml and below, with the highest density (61 colonies) observed at lower meropenem concentrations. In contrast, when ampicillin/sulbactam (8/4 µg/ml) was added, the bacterial growth pattern changed significantly. The highest bacterial count (61 colonies) was observed at a lower colistin concentration (0.125 µg/ml), and there was enhanced inhibition at higher meropenem concentrations (32–64 µg/ml). Similarly, the distribution of colistin’s MIC demonstrated distinctive patterns across different combination therapies. When colistin was combined with meropenem, bacterial growth peaked at meropenem concentrations of 16–32 µg/ml, with the highest density (61 colonies) observed at higher colistin concentrations. When ampicillin/sulbactam (8/4 µg/ml) was added, the bacterial growth pattern shifted notably. The highest bacterial count (61 colonies) was still observed at similar colistin concentrations (16 µg ml^−1^), but there was a more uniform distribution of bacterial growth at moderate colistin concentrations (0.5–8 µg/ml). Additionally, there was enhanced inhibition at lower meropenem concentrations (0–0.125 µg/ml) in the triple-drug combination, demonstrating a synergistic interaction that improved the efficacy of all three antibiotics at concentrations that would be insufficient for bacterial inhibition if used individually.

### Synergy rate of two-drug and three-drug antibiotic combinations

**Fig. 3.**
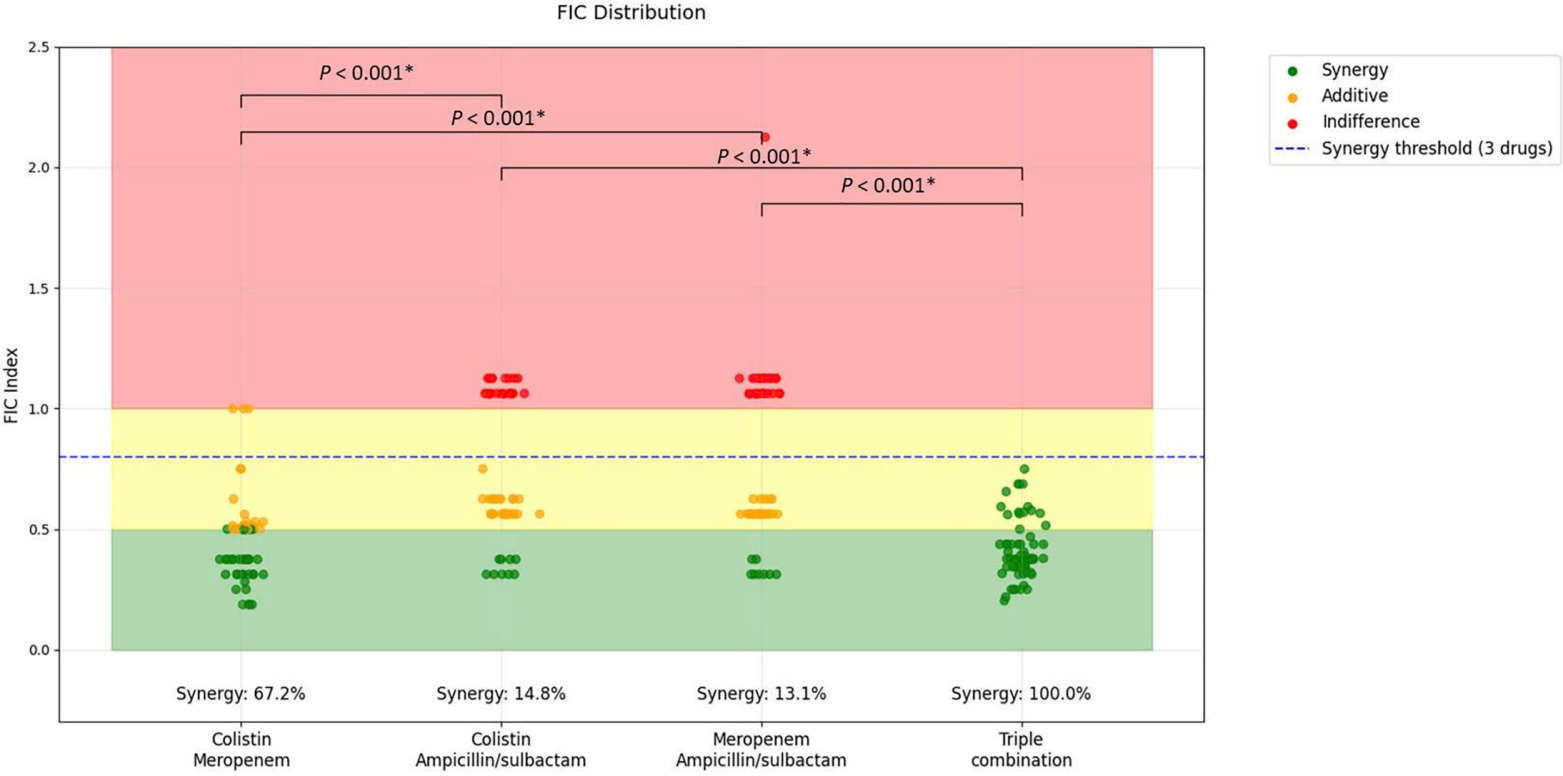
Distribution of FIC values for different antibiotic combinations against CRAB. The coloured zones represent synergy (green), additive (yellow) and indifference (pink) effects. Statistical analysis was performed using the Friedman test followed by Dunn’s post hoc test with Bonferroni correction for multiple comparisons. Asterisks (*) indicate statistical significance with ***P<0.001.

The results of our synergy testing revealed distinctive patterns across the various antibiotic combinations (Fig. 3). None of the combinations demonstrated antagonistic effects (0% across all combinations). The colistin-meropenem combination showed a high synergistic rate of 77% with a 23% additive effect and no indifference. In contrast, both dual combinations containing ampicillin/sulbactam showed lower synergistic rates: 16.4% for colistin-ampicillin/sulbactam and 13.1% for meropenem-ampicillin/sulbactam, with higher rates of additive (50.8 and 41.0%, respectively) and indifferent effects (32.8 and 45.9%, respectively).

Notably, the triple combination of colistin-meropenem-ampicillin/sulbactam achieved a remarkable 100% synergistic rate, demonstrating superior effectiveness compared to all dual combinations. This finding is clearly illustrated in Fig. 3, where the FIC values for the triple combination consistently fall below the 0.8 threshold that defines synergy for three-drug combinations. The clustering of all triple combination FIC values in the synergistic range (green zone) contrasts sharply with the more dispersed distribution of FIC values for the two-drug combinations, spanning across synergistic, additive and indifferent zones.

### Mathematical modelling of antibiotic synergy

**Fig. 4.**
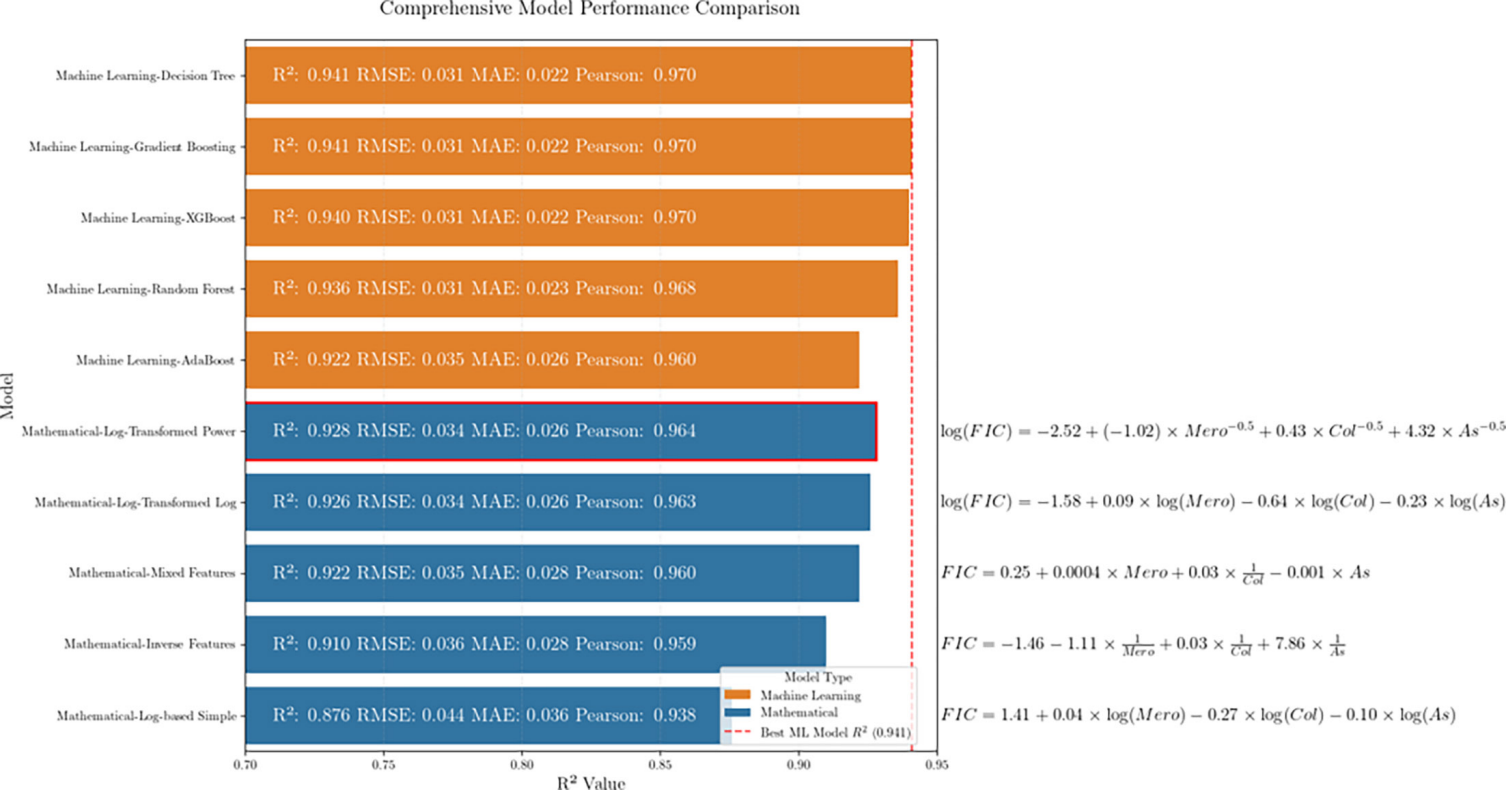
Comprehensive performance comparison of mathematical models and machine learning algorithms for predicting triple antibiotic therapy synergy. Performance metrics (*R*² values) comparing predictive models for triple antibiotic synergy. Orange bars represent machine learning algorithms (decision tree, gradient boosting, XGBoost, random forest and AdaBoost), while blue bars indicate mathematical models (log-transformed power, log-transformed logarithmic, mixed features, inverse features and log-based simple). The log-transformed power model (*R*²=0.928) achieves accuracy comparable to sophisticated machine learning algorithms while providing a transparent, interpretable formula for clinical application.

To develop a predictive model for synergistic interactions, we tested five different mathematical approaches using the separate MIC determinations for colistin, meropenem and ampicillin/sulbactam. The performance metrics for each model were compared against advanced machine learning algorithms to evaluate their relative effectiveness (Fig. 4).

Among the mathematical models, the log-transformed model with power features demonstrated the highest performance, achieving an *R*² value of 0.928, RMSE of 0.034, MAE of 0.026 and Pearson correlation of 0.964. This performance is particularly notable because it approached the predictive capabilities of more complex machine learning algorithms, including machine learning decision tree (*R*²=0.941), machine learning-gradient boosting (*R*²=0.941), machine learning-XGBoost (*R*²=0.940) and machine learning-random forest (*R*²=0.939).

This finding is significant because it demonstrates that a relatively simple, interpretable mathematical formula can achieve prediction accuracy comparable to complex machine learning algorithms that typically require specialized software and expertise to implement. The other mathematical models showed progressively declining performance, with the mathematical-log-based simple model having the lowest *R*² value (0.879) and the highest error metrics.

**Fig. 5.**
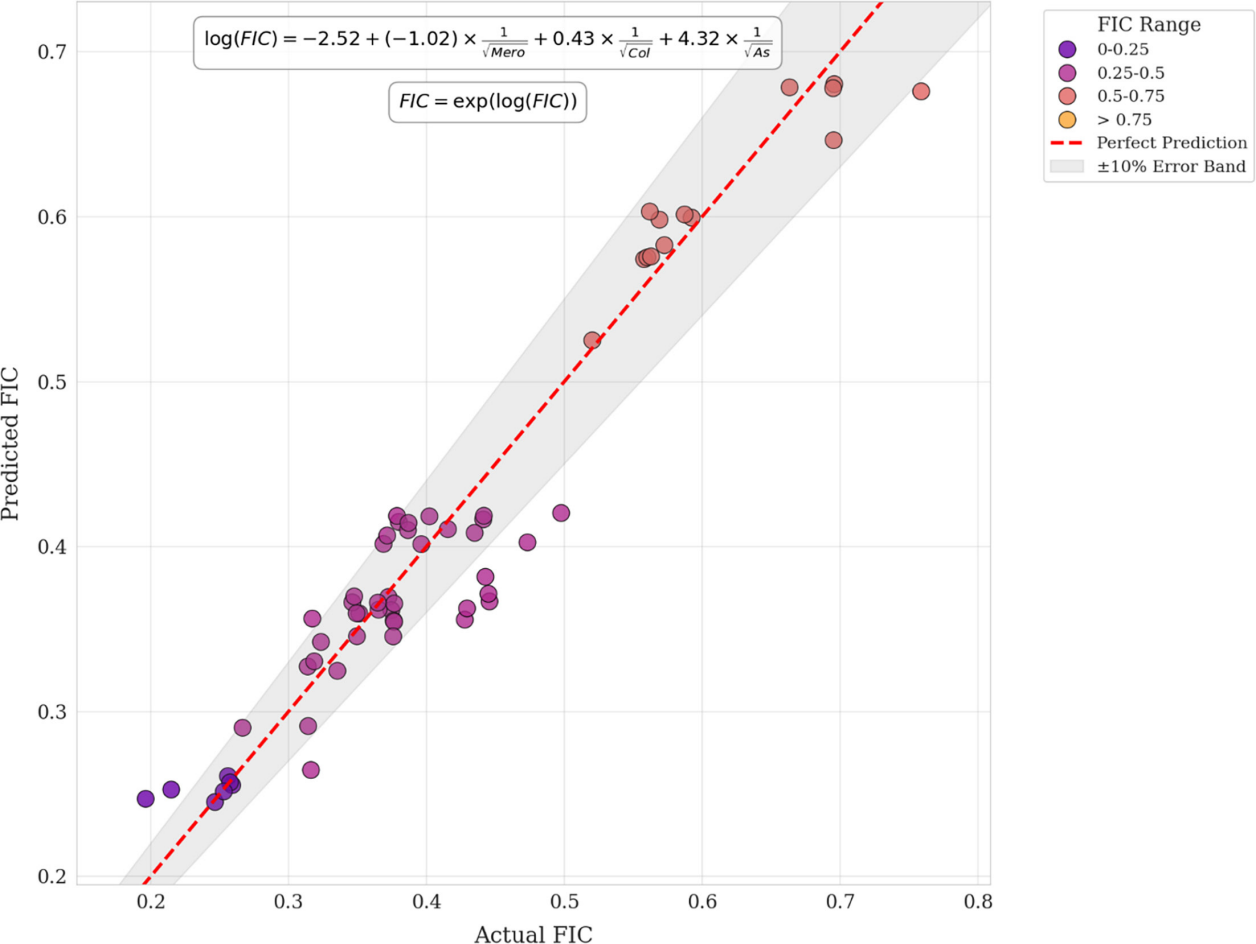
Validation of the log-transformed power model for predicting FIC values. Scatter plot comparing predicted versus actual FIC values using the optimal mathematical model. The equation log(FIC) = −2.52+(−1.02) × 1/√Mero+0.43×1/√Col+4.32×1/√As was used for predictions. Points are colour-coded by FIC range, with the dashed red line representing perfect prediction and the grey band indicating ±10% error margin. The model demonstrates strong predictive accuracy across the range of FIC values, particularly in the synergistic range (0–0.5).

Model validation results (Fig. 5) for the log-transformed model with power features showed good agreement between predicted and actual FIC values. The scatter plot demonstrates that predictions fell primarily within the 10% error band of the actual values. The model exhibited particularly high accuracy in the lower FIC range (0–0.25 and 0.25–0.5), which is critical for correctly identifying synergistic combinations. Some minor deviations were observed in the higher FIC ranges (0.5–0.75), but these represent less clinically significant additive or indifferent interactions.

The mathematical model enables the prediction of FIC values directly from individual MIC results without performing complex checkerboard assays. By inputting the MIC values of colistin, meropenem and ampicillin/sulbactam into the equation, log(FIC)=−2.52+(−1.02)× 1/√Mero+0.43×1/√Col +4.32×1/√As, and then calculating FIC=exp(log(FIC)), clinicians could rapidly determine whether a specific three-drug combination would likely produce synergistic effects for a particular *A. baumannii* isolate. Unlike machine learning models that function as ‘black boxes’, this formula provides a transparent, mechanistically interpretable relationship between individual drug potencies and their synergistic interactions, potentially offering insights into the underlying pharmacological principles governing antibiotic synergy.

### Combined MIC analysis and clinical achievability

The combined MIC values achieved through the triple combination ranged from 0.0625/4/4 to 0.25/16/4 µg ml^−1^ for colistin/meropenem/ampicillin-sulbactam, respectively. When compared to established pharmacokinetic data, these concentrations fall within clinically achievable ranges. For colistin, the combined MIC values (0.0625–0.25 µg ml^−1^) are well below the typical steady-state plasma concentrations of 2–4 µg ml^−1^ achieved with standard dosing regimens. Similarly, meropenem concentrations of 4–16 µg ml^−1^ in the combination are attainable with standard dosing (1–2 g every 8 h), considering that free drug concentrations of 20–40 µg ml^−1^ are commonly achieved. The ampicillin/sulbactam component at 4 µg ml^−1^ corresponds to the standard CLSI breakpoint concentration, indicating therapeutic feasibility at conventional dosing regimens.

## Discussion

### Patient and specimen characteristics

In our study, the 53 patients had a mean age of 72.3 years, which is higher than the mean ages reported in many other *A. baumannii* infections studies, typically ranging from 50 to 65 years [6,22]. This discrepancy may be attributed to the demographic characteristics of the patient population served by our institution, with a higher proportion of elderly individuals. Alternatively, it may reflect a particular vulnerability of older adults to CRAB infections in our setting. It is known that advanced age is associated with immunosenescence, a greater burden of comorbidities and increased exposure to healthcare interventions, all of which contribute to a heightened susceptibility to resistant pathogens. Moreover, the patients were predominantly male and were hospitalized primarily in the Intensive Care Unit, followed by Internal Medicine and Surgery. The predominance of elderly male patients in our cohort aligns with previous epidemiological studies of *A. baumannii* infections. For instance, Teerawattanapong *et al*. reported that *A. baumannii* infections in Southeast Asia disproportionately affect older adults, with some studies indicating a male predominance of up to 70% [4]. The high representation of males in our cohort may be attributed to gender-related differences in comorbidity profiles, healthcare-seeking behaviours and possibly smoking history, which is a known risk factor for respiratory infections [23]. Among these patients, the most common diagnosis was pneumonia, followed by sepsis and wound infection. This distribution reflects the typical clinical presentations of *A. baumannii* infections reported in the literature, with respiratory tract infections consistently representing the most frequent manifestation [24]. The high prevalence of pneumonia in our study correlates with the predominance of respiratory specimens, with sputum samples constituting 80.0% of all specimens collected. This strong association between specimen type and clinical presentation supports the pathogenic role of *A. baumannii* in these infections rather than mere colonization. The high proportion of intensive care unit (ICU) patients (54.7%) in our study is consistent with the established risk profile for *A. baumannii* infections, as intensive care settings entail multiple risk factors, including mechanical ventilation, multiple invasive procedures, broad-spectrum antimicrobial use and prolonged hospitalization [25]. This setting also explains the substantial prevalence of eXDR isolates in our collection, as ICUs often serve as reservoirs for the most resistant pathogens due to extensive antimicrobial selective pressure.

Regarding the specimen types, our specimen distribution differed somewhat from other CRAB studies. While respiratory specimens were most common in our cohort, followed by blood and urine, some international study reports describe higher proportions of wound and blood isolates [26,27]. This variation might be attributed to differences in institutional infection control practices, patient populations or regional prevalence of specific *A. baumannii* clones with varying tissue tropism. Notably, the relatively low rate of bloodstream infections in our cohort despite a 35.8% incidence of sepsis suggests that many sepsis cases may have been secondary to primary respiratory infections rather than primary bacteraemia.

The patient demographics and clinical characteristics observed in our study support the generalizability of our findings to similar healthcare settings, particularly tertiary care centres with high acuity patients. However, the predominance of elderly patients may limit the direct applicability of our antimicrobial synergy findings to younger populations, who might exhibit different pharmacokinetic profiles and tolerance to the evaluated antibiotic combinations. Similarly, the high proportion of respiratory infections in our cohort should prompt caution when extrapolating our results to other types of *A. baumannii* infections, such as those affecting the central nervous system or deep-seated abscesses, where antibiotic penetration and efficacy may differ substantially.

### Comparison of MIC effects of drugs used alone and combinations

Our antimicrobial susceptibility testing revealed distinct activity patterns for colistin, meropenem and ampicillin/sulbactam against carbapenem-resistant *A. baumannii* isolates. Colistin demonstrated the highest activity, with all isolates exhibiting very low MICs ranging from 0.125 to 0.25 µg ml^−1^, which is well below the CLSI resistance breakpoint of 2 µg ml^−1^, indicating universal non-resistance to colistin in our collection. According to current CLSI guidelines, colistin susceptibility is classed only as ‘intermediate’ or ‘resistant’, with no ‘susceptible’ designation [19]. In stark contrast, meropenem showed poor activity, with all isolates displaying high-level resistance. MICs ranged from 32 to 64 µg ml^−1^, with some exceeding 128 µg ml^−1^. These values are significantly above the CLSI breakpoint of 8 µg ml^−1^, confirming a truly carbapenem-resistant population [19]. Like meropenem, ampicillin/sulbactam demonstrated limited efficacy, with elevated MIC ranging from 32/16 to 128/64 μg ml^−1^.

Regarding the carbapenem resistance observed in *A. baumannii* strains isolated in our study, all isolates exhibited a high level of resistance, consistent with alarming global trends. Worldwide, carbapenem resistance has become a critical issue in healthcare-related infections, with resistance rates spanning 40–70% across numerous countries and mortality rates varying from 30% to 75% among affected patients [2,3]. In Southeast Asia, the situation is even more severe, with resistance rates exceeding 65% in many countries [4]. Our finding of 100% carbapenem resistance aligns with previous Vietnamese studies reporting resistance rates ranging from 55 to 90% [6,28], highlighting the particularly challenging antimicrobial resistance landscape in this region. Interestingly, our isolates retained susceptibility to colistin, which contrasts with the global trends of gradually increasing resistance. A recent meta-analysis indicated that colistin resistance in *A. baumannii* has doubled from ~2% in 2011 to 4% in recent years [29]. However, regional variations are notable, with some centres in Asia reporting resistance rates as high as 30% [30]. The preserved susceptibility in our study likely reflects local antimicrobial stewardship practices, particularly the restricted use of colistin as a last-resort therapy. Meanwhile, the high rate of ampicillin/sulbactam resistance (98.4%) in these isolates exceeds global averages, but it aligns with patterns observed in regions with elevated carbapenem resistance. For instance, studies from China and Turkey have reported similar ampicillin/sulbactam resistance rates of 80–90% among carbapenem-resistant isolates [31,32]. This correlation between carbapenem and ampicillin/sulbactam resistance suggests potential shared resistance mechanisms or co-selection pressure due to similar clinical use patterns. Colistin’s preserved activity has important therapeutic implications. As a polymyxin antibiotic, colistin disrupts the bacterial outer membrane by displacing divalent cations from LPS molecules, increasing membrane permeability [14]. This unique mechanism partly explains its efficacy against carbapenem-resistant isolates. However, colistin monotherapy has been linked with suboptimal clinical outcomes and the risk of rapid resistance emergence during treatment [33], emphasizing the need for combination therapies in managing these challenging infections.

### Synergy rates of two-drug and three-drug antibiotic combinations

Our synergy testing revealed distinctive patterns across different antibiotic combinations against carbapenem-resistant *A. baumannii*. Among dual combinations, colistin-meropenem exhibited the highest synergy rate (77%), with the remaining 23% showing additive effects. Notably, no indifferent or antagonistic interactions were observed. This finding is particularly significant given the high-level meropenem resistance (MICs ranging from 32 to 512 µg ml^−1^), suggesting that colistin can enhance meropenem activity even against highly resistant strains. In contrast, dual combinations involving ampicillin/sulbactam showed considerably lower synergy rates: 16.4% for colistin-ampicillin/sulbactam and 13.1% for meropenem-ampicillin/sulbactam. These combinations are predominantly additive (50.8% and 41.0%, respectively) or indifferent (32.8% and 45.9%, respectively). The substantial difference in synergy rates underscores the specific mechanistic compatibility between colistin’s membrane-disrupting properties and meropenem’s inhibition of cell wall synthesis as mentioned in Huang *et al*. [34]. The most striking result was the exceptional 100% synergy rate achieved by the triple combination of colistin-meropenem-ampicillin/sulbactam. All tested isolates exhibited FIC indices consistently below the 0.8 threshold defining synergy for three-drug combinations, with most values clustered in the 0.3–0.6 range. This universal synergy across a genetically diverse collection suggests that this triple combination targets essential bacterial processes that are not simultaneously protected by existing resistance mechanisms.

Our findings align with and extend previous research on antibiotic synergy against *A. baumannii*. The 77% synergy rate observed for colistin-meropenem exceeds those reports in earlier studies. Wang *et al*. reported 47% synergy in isolates from China [35], while Ramadan *et al*. found a 63.6% synergy rate in Egypt [36]. The higher synergy rates in our study might reflect regional differences in strain genotypes or resistance mechanisms. Up to now, data on three-drug combinations remain limited. Lenhard *et al*. demonstrated enhanced bacterial killing with ampicillin-sulbactam-based combinations in a hollow-fibre infection model, though they did not achieve the universal synergy observed in our study [15]. A key aspect of our findings is that synergy was achieved using ampicillin/sulbactam at the standard CLSI breakpoint concentration (8/4 µg ml^−1^), rather than the high doses (equivalent to 6–9 g day^−1^) typically recommended for *A. baumannii* infections. This suggests that effective treatment may be possible at standard doses when part of an optimized combination regimen, potentially reducing the risks of toxicity associated with high-dose regimens. Nephrotoxicity occurs in 30–60% of patients receiving polymyxins, while high-dose *β*-lactams have been associated with neurotoxicity and seizures in up to 15% of critically ill patients [11]. Demonstrating synergy at standard doses offers a promising path toward effective therapy with a reduced toxicity burden. These findings suggest several important modifications for current treatment strategies for carbapenem-resistant *A. baumannii* infections. First, they support consideration of triple-drug therapy for severe infections caused by XDR isolates, particularly when standard dual combinations may be inadequate. Second, they indicate that standard dosages of ampicillin/sulbactam may be effective in triple combinations, potentially allowing dose reduction from the currently recommended high-dose regimens. Additionally, our results support the value of synergy testing in guiding individualized therapy for difficult-to-treat infections. Integration of our predictive mathematical model could further refine this approach by allowing rapid determination of potentially synergistic combinations without the need for labour-intensive laboratory testing. While promising, these *in vitro* findings require validation in animal models and clinical studies before widespread implementation. Nevertheless, they provide a strong foundation for future research and highlight a potential pathway to optimize therapy for one of the most challenging MDR pathogens in modern healthcare.

### Mathematical modelling of antibiotic synergy

Our log-transformed power model showed strong predictive performance for FIC values (*R*² = 0.928, RMSE=0.034 and Pearson *r*=0.964), described by the following equation: log(FIC)=−2.52+(−1.02)× 1/√Mero+0.43×1/√Col+4.32×1/√As. The inverse square root transformation (1/√X) captures the non-linear relationship between antibiotic concentrations and synergistic effects, compressing differences at high MIC and amplifying them at low MIC, where efficacy shifts are impactful [37]. Coefficient values reveal that higher meropenem MIC reduce synergy, confirming that meropenem plays a less significant role in the synergistic mechanism when bacteria exhibit high-level resistance to it. This mathematically aligns with the biological understanding that highly resistant strains derive less benefit from meropenem in the combination. Colistin’s positive coefficient indicates that reduced susceptibility may enhance synergy, possibly due to its membrane-permeabilizing role. The large coefficient for ampicillin/sulbactam indicates its dominant contribution to synergy, supporting its primary bactericidal role in this combination [10,12]. Overall, the model highlights ampicillin/sulbactam as the key driver of synergy, followed by colistin, with meropenem playing a minor role. This aligns with previous studies showing sulbactam’s unique efficacy against *A. baumannii* through its binding to PBPs [10], while suggesting that meropenem’s contribution diminishes significantly in highly resistant isolates. The model further suggests that optimal synergy would be expected in isolates with lower meropenem MICs, higher colistin MICs (within the susceptible range) and higher ampicillin/sulbactam MICs. This mathematical prediction could guide clinical application of this combination therapy, particularly for isolates with varying resistance profiles.

### Clinical implications, limitations and future directions

Our findings have important clinical implications. The triple combination of colistin-meropenem-ampicillin/sulbactam, administered at standard dosages, demonstrated 100% synergy across all CRAB isolates when using ampicillin/sulbactam at the standard CLSI breakpoint concentration (8/4 µg ml^−1^). This approach avoids the need for high doses (6–9 g day^−1^) and may reduce the risk of toxicity [11]. The proposed mathematical model represents a paradigm shift from traditional synergy testing toward the rapid prediction of antibiotic interactions. Using routine MIC determinations and our equation, log(FIC) = −2.52+(−1.02) × 1/√Mero+0.43×1/√Col+4.32×1/√As, laboratory personnel can calculate FIC values within minutes. FIC values <0.8 predict synergistic interactions, enabling clinicians to prescribe the triple combination at standard dosages rather than empirically escalating to high-dose regimens. This model can be integrated into laboratory information systems to provide real-time synergy predictions, thereby supporting antimicrobial stewardship. However, several limitations must be acknowledged. A key limitation is that our predictive model was developed exclusively with isolates that demonstrated universal synergy (100% synergistic rate). Consequently, its ability to predict indifferent or antagonistic interactions remains untested, as no isolates in our cohort exhibited non-synergistic patterns. This represents a critical knowledge gap that must be addressed through validation with isolates showing a broader range of interaction profiles, including additive, indifferent and antagonistic combinations. Additionally, the relationship between *in vitro* MIC determinations and actual tissue/plasma concentrations varies by infection site and patient pharmacokinetics, limiting direct extrapolation to all clinical scenarios. Our cohort predominantly consisted of respiratory specimens (80.0%), with limited representation from bloodstream infections, urinary tract infections and wound infections. Antibiotic efficacy and synergistic interactions may differ across infection sites due to variations in local pharmacokinetics, biofilm formation potential and host immune factors. Finally, clinical implementation faces key barriers such as limited workforce capacity with insufficient expertise in mathematical modelling and computational tools, poor integration between microbiology laboratories and clinical staff and resource constraints for investing in computational infrastructure and staff training [38].

This methodology provides a framework for predictive models of other antibiotic combinations, supporting the development of ‘synergy prediction libraries’ adapted to local resistance patterns. In Vietnam, where *Klebsiella pneumoniae* shows high carbapenem resistance (27%) with a high prevalence of extended-spectrum β-lactamase production (90.9%), and *A. baumannii* exhibits universal carbapenem resistance, extending this approach to other ESKAPE pathogens could transform antimicrobial stewardship [39]. Further validation studies are therefore essential to confirm the model’s applicability across diverse clinical specimens and infection types. Clinical trials comparing standard versus high-dose triple therapy are also necessary to confirm therapeutic benefits and to guide rationalized combination therapy strategies.

## Conclusion

In conclusion, our study demonstrates that the triple combination of colistin, meropenem and ampicillin/sulbactam exhibits consistent synergistic activity against carbapenem-resistant *A. baumannii*, even when using standard ampicillin/sulbactam concentrations aligned with CLSI breakpoints. Furthermore, we have developed a predictive mathematical model based on MIC values, enabling accurate identification of synergistic combination therapy without the need for labour-intensive laboratory testing. This integrated approach, combining *in vitro* synergy testing with predictive modelling, marks a significant step forward in antimicrobial stewardship for MDR infections. By allowing synergy prediction from routine susceptibility data, it offers a practical tool to optimize therapy, improve clinical outcomes and minimize reliance on empirical or potential toxic treatment regimens.

### Guidelines and regulations

This research strictly adhered to the CLSI guidelines for antimicrobial susceptibility testing (CLSI M100-ED33, 2023). All laboratory procedures complied with the Vietnamese Ministry of Health’s regulations including Circular No. 37/2017/TT-BYT dated 25 September 2017, on ‘Regulations on biosafety practices in laboratories’ and Circular No. 16/2018/TT-BYT dated 20 July 2018, on ‘Regulations on infection control in medical examination and treatment facilities’. The microbiological testing procedures followed the technical guidelines outlined in Decision No. 6769/QĐ-BYT dated 8 November 2018, by the Minister of Health on ‘Technical procedure guidelines for microbiology specialty’.

All antimicrobial susceptibility testing protocols, including broth microdilution and checkerboard assays, were performed in accordance with standardized procedures outlined in CLSI document M07 ‘Methods for Dilution Antimicrobial Susceptibility Tests for Bacteria That Grow Aerobically’. Quality control was conducted using reference strains *P. aeruginosa* ATCC 27853 and *E. coli* ATCC 25922 as specified in CLSI guidelines. The study ensured compliance with both national and international standards for microbiological testing and antimicrobial resistance surveillance.

## Abbreviations

CAMHB: cation-adjusted Mueller-Hinton broth
CLSI: Clinical and Laboratory Standards Institute
CRAB: carbapenem-resistant *Acinetobacter baumannii*
FIC: fractional inhibitory concentration
ICU: intensive care unit
MAE: mean absolute error
MDR: multidrug-resistant
PBP: penicillin-binding protein
PDR: pandrug-resistant
RMSE: root mean square error
XDR: extensively drug-resistant
